# Expression, purification, and initial characterization of different domains of recombinant mouse 2',3'-cyclic nucleotide 3'-phosphodiesterase, an enigmatic enzyme from the myelin sheath

**DOI:** 10.1186/1756-0500-3-12

**Published:** 2010-01-21

**Authors:** Matti Myllykoski, Petri Kursula

**Affiliations:** 1 Department of Biochemistry, University of Oulu, Oulu, Finland; 2 Centre for Structural Systems Biology (CSSB), Helmholtz Centre for Infection Research (HZI), German Electron Synchrotron (DESY), Hamburg, Germany

## Abstract

**Background:**

2',3'-cyclic nucleotide 3'-phosphodiesterase (CNPase) is an enigmatic enzyme specifically expressed at high levels in the vertebrate myelin sheath, whose function and physiological substrates are unknown. The protein consists of two domains: an uncharacterized N-terminal domain with little homology to other proteins, and a C-terminal phosphodiesterase domain.

**Findings:**

In order to be able to fully characterize CNPase structurally and functionally, we have set up expression systems for different domains of CNPase, using a total of 18 different expression constructs. CNPase was expressed in *E. coli *with a TEV-cleavable His-tag. Enzymatic activity assays indicated that the purified proteins were active and correctly folded. The folding of both the full-length protein, as well as the N- and C-terminal domains, was also studied by synchrotron CD spectroscopy. A thermal shift assay was used to optimize buffer compositions to be used during purification and storage. The assay also indicated that CNPase was most stable at a pH of 5.5, and could be significantly stabilized by high salt concentrations.

**Conclusions:**

We have been able to express and purify recombinantly several different domains of CNPase, including the isolated N-terminal domain, which is folded mainly into a β-sheet structure. The expression system can be used as an efficient tool to elucidate the role of CNPase in the myelin sheath.

## Background

The myelin sheath is a membrane structure present in the vertebrate nervous system, formed when a glial cell wraps its plasma membrane several times around the axon. Myelin facilitates the transmission of nerve impulses, leading to up to 100 times faster impulse velocity in myelinated vs. non-myelinated axons. This speed is necessary for the proper functioning of the nervous system, and loss of myelin leads invariably to severe complications. Myelin consists of tightly packed lipid layers with relatively little water. This is probably a significant factor in that the protein composition of myelin is unique. Specific properties of myelin proteins often include hydrophobicity and attachment to membranes [1]. Thus, their recombinant expression and purification are not trivial.

2',3'-cyclic nucleotide 3'-phosphodiesterase (CNPase) is an enzyme expressed abundantly in myelin. In myelin, CNPase is localized in the non-compact regions, such as the periaxonal membrane and the paranodal regions [2]. It forms up to 4% of protein in the central nervous system myelin and up to 0.4% in the peripheral nervous system; thus, it is the most abundant protein in the non-compacted myelin membrane. A post-translational isoprenylation at a cysteine residue close to the C terminus makes it possible for the protein to attach itself to membranes [3,4]. While myelin in CNPase-deficient mice [5] appeared normal, they develop progressive axonal neurodegenerative disease. The mutants have also indicated roles for CNPase in axoglial interactions at the nodes of Ranvier [6], and in proper myelination of small-diameter axons [7]. CNPase apparently takes part in both myelination and in a signal transduction cascade between myelin and the axon [8]. CNPase has also been implicated as a potential antigen and marker in autoimmune disease [9-11].

The enzymatic activity of CNPase, the hydrolysis of 2',3'-cyclic nucleotides to 2'-nucleotides, was the basis of the discovery of the enzyme from brain tissue in the 1960s [12]. The activity is specific to 2',3'-cyclic nucleotides, and the enzyme cannot hydrolyze 3',5'-cyclic adenosine monophosphate [13]. The physiological substrate for the enzymatic reaction of CNPase has been a mystery, because 2',3'-cyclic nucleotides have not been discovered *in vivo*. However, recently, components of RNA metabolism have been suggested to be the *in vivo *substrate. Mammalian CNPase was shown to be able to fill in for the yeast tRNA ligase in tRNA repair [14], and it was shown to bind mRNA in cultured oligodendrocytes and inhibit translation *in vitro *[15].

CNPase is expressed as two isoforms, produced from the same gene. The 20-residue extension in the N-terminus of CNPase 2 functions as a mitochondrial targeting signal that is controlled by phosphorylation [16]. The full-length CNPase can be divided into two folded domains. The C-terminal domain contains the phosphodiesterase activity [13,17], and is, hence, usually termed the catalytic domain. The crystal structure of the C-terminal domain with residues 166-379 of human CNPase has been determined [18]. Along with NMR data [19], these results confirm that CNPase belongs to the 2H phosphoesterase superfamily of proteins. This superfamily is characterized by two His - X - Thr motifs, forming the core of the active site. Many proteins in this superfamily have functions related to RNA.

The N-terminal domain, approximately the first 180 residues, of CNPase is very poorly characterized, and its purification has not previously been reported. It is not necessary for the phosphodiesterase activity *in vitro *[17]. The N-terminal region does, however, contain an ATP or GTP binding motif, the P-loop [20], and has a low homology to the family of P-loop containing nucleotide triphosphate hydrolases. Recently, binding of purine nucleotides and weak hydrolysis of purine triphosphates by CNPase was detected [21], but it was not experimentally proven whether the binding and hydrolysis occur in the N- or the C-terminal domain. The availability of the purified N-terminal domain from CNPase would be required to fully characterize such functions.

Here, we have set up an expression system for different domains of mouse CNPase in *E. coli*. Constructs for both the N- and C-terminal regions of CNPase and the full-length protein have been prepared. Several constructs of different length have been prepared; having multiple constructs for the same protein increases significantly the chance of obtaining soluble protein and, ultimately, well-diffracting crystals [22]. A number of the CNPase constructs have been used successfully for large-scale protein expression and purification, and initial characterizations are reported here, including assays for activity, stability, and folding of the different domains.

## Materials and methods

### Vector construction

Details of the vector construction are in Additional File 1. Briefly, the Gateway system was used to subclone inserts encoding mouse CNPase [23] into pTH27 [24]. The resulting constructs express recombinant CNPase domains with an N-terminal His tag, removable by TEV protease [25].

### Expression and purification

For large-scale expression, plasmids were transformed into *E. coli *Rosetta (DE3). A transformed colony was picked into 10 ml LB medium with 100 μg/ml ampicillin and 34 μg/ml chloramphenicol. The culture was grown overnight at +37°C, and 1-10 ml of it was used to start the large-scale culture (1000 ml) on the following day.

For the C-terminal domain of CNPase, the large-scale culture was grown and induced at +37°C. The culture was grown with shaking at +37°C, until the optical density at 600 nm was 0.4-0.6. Protein expression was induced with the addition of isopropyl β-D-1-thiogalactopyranoside (IPTG) to 0.2 mM, and the culture was incubated at +37°C for 3 h. The cells were collected by centrifugation and resuspended in lysis buffer (50 mM Na-Phosphate pH 7.5, 500 mM NaCl, 10% Glycerol and 1 mM DTT). Cells were flash-frozen in liquid nitrogen and stored at -70°C until use.

For the large-scale expression of the N-terminal domain of CNPase, the autoinduction medium ZYM-5052 containing the appropriate antibiotics was used [26]. The culture was shaken at 18°C for approximately 40 h, and the cells were collected and resuspended in lysis buffer (50 mM Tris-HCl pH 8, 500 mM NaCl, 10% Glycerol, 5 mM CaCl_2 _and 1 mM DTT). The cell suspension was frozen and stored at -70°C.

The large-scale expression of the full-length protein was initially done in a similar fashion to the C-terminal domain, with the temperature for induction lowered to 18 - 20°C. Also, a similar autoinduction method to the one used for the N-terminal domain was used, with the exception that the buffer component of the lysis buffer was HEPES at pH 7.

To lyse the cells, lysozyme was added to 0.1 mg/ml; the cell suspension was then sonicated repeatedly, until it appeared non-viscous. Cell debris was removed by centrifugation at 27000 g for 30 min. The supernatant was applied to a Ni-TED (Macherey-Nagel) column. The column was washed with lysis buffer until the baseline at 280 nm was stable and elution was performed with lysis buffer including 250 mM imidazole. TEV protease [25] was added to the sample, and the protein was dialyzed overnight back into the lysis buffer (without imidazole). The dialyzed protein was applied back to the Ni-TED column, and the flow-through was collected.

The sample was concentrated to 2 ml and applied to a Hiload 16/60 Superdex 200 or a Hiload 16/60 Superdex 75 column (GE Healthcare). The protein-containing peaks were analyzed by SDS-PAGE, and the fractions containing CNPase were pooled, concentrated, and stored on ice until further use.

### Thermal shift assay

The thermal shift assay was done similarly to a previous report [27]. Measurements were made with the 7500 Real-Time PCR System, using 96-Well Optical Reaction Plates (Applied Biosystems). Each well contained 1-5 μg of the protein and 1-2 X SYPRO Orange dye (Molecular Probes). The temperature was elevated steadily in 1°C steps from 21°C to 90°C. The fluorescence of the SYPRO Orange probe was measured using excitation at 490 nm and emission at 575 nm. Measurements were done in duplicate or triplicate.

### Enzymatic activity assays

Kinetic measurements were made as previously described [17]. In this method, CNPase cleaves the phosphodiester bond in 2', 3'-cyclic NADP (cNADP) [28]. NADP is then reduced to NADPH by glucose-6-phosphate dehydrogenase, to transform glucose-6-phosphate to 6-phosphogluconolactone. The amount of formed NADPH is directly relative to the activity of CNPase and can be measured spectrophotometrically at 340 nm with the absorption coefficient 6.22 cm^-1 ^mM^-1^.

The reaction mixture contained 100 mM MES buffer at pH 6, 30 mM MgCl_2_, 5 mM glucose-6-phosphate (Sigma) and 0,6 U glucose-6-phosphate dehydrogenase (Sigma). The concentration of the substrate cNADP (Sigma) was 2, 1, 0.5, 0.2, 0.1, 0.05, 0.02 and 0 mM. 500 or 5000 ng of CNPase were used. The measurements were performed at 25°C using 96-Well UV-Star Microplates (Greiner Bio-One) with the Powerwave X device (BIO-TEK Instruments Inc.) and KC Junior software (BIO-TEK Instruments Inc.).

### Synchrotron radiation CD spectroscopy

The folding of the full-length CNPase and the N- and C-terminal domains was studied by SRCD at the CD1 beamline of the ASTRID storage ring, ISA, Århus (Denmark). Prior to measurement, the proteins were dialyzed into a buffer suitable for SRCD analysis (50 mM sodium phosphate, 500 mM NaF, 10% glycerol, 1 mM DTT; pH either 6 (full-length and C-terminal CNPase) or 8 (N-terminal domain)). CD spectra were collected in the wavelength range of 170-280 nm. The protein concentrations were 0.5 (full-length), 1.1 (N-terminal), and 4.7 (C-terminal) mg/ml; the pathlength was either 100 μm (full-length and N-terminal) or 18 μm (C-terminal). The C-terminal domain was also studied in the presence of 1 M ammonium sulfate. The spectra were processed with CDtool [29] and analyzed using Dichroweb [30].

## Results and discussion

### Vector construction

**Figure 1:**
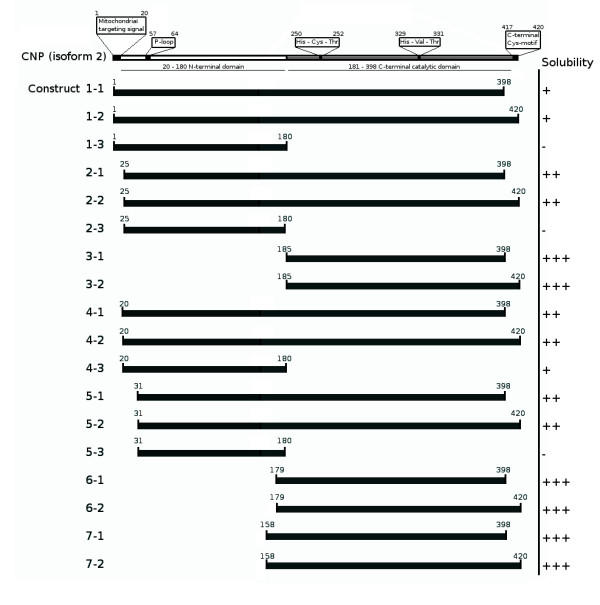
**A schematic of the main characteristics of the CNPase protein and of the length and relative solubility of the prepared constructs**.

A total of 18 expression clones were prepared for mouse CNPase, as shown in Figure 1 and Additional File 2. Eight constructs were made for the full-length protein, six for the C-terminal domain, and four for the N-terminal domain. All constructs can be used to express protein that has an N-terminal hexahistidine tag, followed by a TEV protease cleavage site and the corresponding CNPase domain. The entry clones can also be used to generate other expression vectors in the Gateway system, such as those used in insect cells.

### Expression & purification

**Figure 2:**
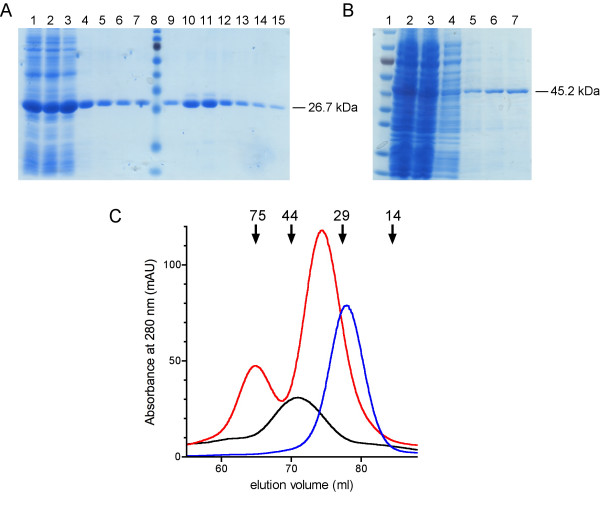
**Purification of recombinant CNPase**. A. SDS-PAGE from a Ni-TED purification of a C-terminal CNPase construct. 1, soluble lysate; 2, flow-through; 3-7, washes; 8, marker; 9-15, imidazole elutions. The calculated size for the recombinant protein was 26.7 kDa. B. SDS-PAGE from a Ni-TED purification of a full-length CNPase construct. 1, marker; 2, soluble lysate; 3, flow-through; 4, wash; 5-7, elutions. The calculated size of the recombinant protein was 45.2 kDa. The gels in A and B were stained with PageBlue Protein Staining Solution (Fermentas). The molecular weight marker in both gels is the PageRuler Prestained Protein Ladder, with sizes (from bottom to top) 10, 15, 25, 35, 40, 55, 70, 100, and 130 kDa. C. Size exclusion chromatograms (on a HiLoad 16/60 Superdex 200 column) for different CNPase constructs. The black line is a chromatogram for a full-length CNPase (construct 2-1), the red line for the catalytic domain containing the C-terminal tail (construct 6-2), and the blue line for the catalytic domain without the C-terminal tail (construct 6-1). The absorbance of the catalytic domain without the C-terminal tail was scaled to 10% for it to be in comparable scale. Note the dimer seen in the presence of the C-terminal tail. The elution volumes of molecular weight markers (in kDa) are indicated above the graph.

Several constructs were successfully used for protein expression. The C-terminal constructs were particularly simple to express in large amounts; the amount of recombinant protein in the lysate clearly exceeds the binding capability of the column (Figure 2A). Consequently, the purity reached with a single purification step was good. TEV protease cleavage did not work for the C-terminal domain constructs prepared with forward primer 3, possibly due to steric hindrance in the folded protein. Thus, further expression of the catalytic domain was done with constructs prepared with forward primer 6 (6 amino acid residues longer in the N-terminus), with which the TEV cleavage worked.

The full-length constructs had to be expressed at lower temperatures in order to have the protein expressed in soluble form, and less soluble protein was obtained. The purification of the full-length protein was done in a similar fashion to the C-terminal domain (Figure 2B).

The expression and purification of the N-terminal domain proved to be a much tougher task. Only one construct (residues 20-180) out of the four that were produced could be used to express the protein in soluble form; when expressed with the autoinduction method, and even then, only in small amounts. The reduced expression level also makes the purification of the N-terminal domain of CNPase more difficult.

All samples initially purified by Ni-affinity chromatography were further purified with size exclusion chromatography (Figure 2C). The constructs that contain the very C-terminal extension (residues 399-420) tend to form stable dimers during purification. This is probably due to the cysteine residue 417, which is part of an unfolded tail. In fact, the observed dimers could be broken into monomers by DTT treatment (data not shown). *In vivo*, this cysteine is implicated in isoprenylation [20].

The typical yield of purified protein from 1000 ml of culture for the C-terminal constructs was tens of milligrams. For the full-length constructs, the yield was milligrams and for the N-terminal constructs much less. The purified constructs are now being used for biochemical characterizations and crystallization, in order to determine the structure-function relationships in CNPase.

### Thermal shift assay

**Figure 3:**
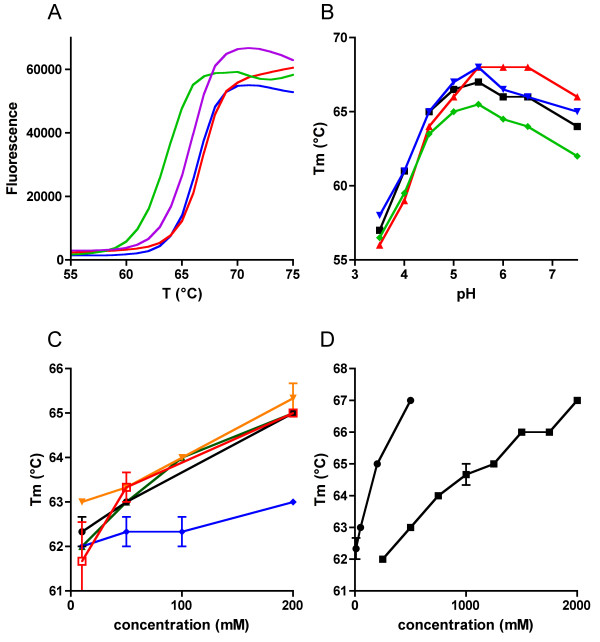
**Graphical representation of the melting temperatures defined by the thermal shift assay**. A. A selection of thermal shift assay melting curves for the CNPase catalytic domain in pH 5.5 (0 mM NaCl, blue; 500 mM NaCl, red) and 7.5 (0 mM NaCl, green; 500 mM NaCl, purple). A clear increase in the melting temperature can be seen from the graphs, induced by both pH 5.5 and high salt concentration. B. The stability of the C-terminal domain in different conditions as a function of pH. Black, 150 mM NaCl; red, 500 mM NaCl; blue, 10% glycerol; green, 5% PEG400. C. The effect of different salts on the stability of the full-length protein (construct 4-1). Blue, sodium acetate; red, lithium sulfate; black, ammonium sulfate; green, sodium phosphate; orange, sodium citrate. The buffer in the experiment was 50 mM Bis-Tris, pH 5.5. D. A comparison between ammonium sulfate (circles) and sodium chloride (squares). See Additional File 3 for more details on the thermal shift assays.

Initially, all buffers used in the purification were of near-neutral pH with a low NaCl and glycerol content. The solubility and stability of the protein did not appear to be ideal under these conditions, as evidenced by precipitation during storage. The apparent melting temperatures (T_m_) for full-length and C-terminal constructs were determined under various conditions (Figure 3) using a thermal shift assay, in the presence of SYPRO Orange, which binds to the hydrophobic internal parts of the unfolding protein and produces a rise in fluorescence upon protein denaturation. When applied in the purification protocols, the conditions with higher T_m_values were found to correlate with increased solubility and stability of the proteins. In pH 5.5 and in the presence of 500 mM NaCl, CNPase is much more stable than at neutral pH with lower salt content. For example, the protein could be stored on ice at +4°C for weeks in the optimized buffer. A rather general stabilization of the protein was observed with different salts (Figure 3C, D). The melting temperature and stability during purification are both also consistently higher for the catalytic domain than for the full-length protein. A clear correlation exists between the apparent melting temperature obtained from the assay and the behaviour of the protein during preparation and storage.  See Additional file 3 for more details.

### Enzymatic activity measurements

The phosphodiesterase activity of CNPase was assayed with a coupled enzyme assay [17,28]. Activity measurements were made for two different full-length constructs and two C-terminal domain constructs (Table 1). The results clearly indicate that all tested constructs were active in the phosphodiesterase reaction. The activity was similar to that seen in earlier studies. Furthermore, the C-terminal tail (22 residues) has no significant effect on the catalytic activity; neither do the first 24 N-terminal residues of the full-length protein.

**Table 1:** Enzymatic characterization

	CNPase construct				**Lee *et al.***[19]	
	1-1 (500 ng)	2-1 (5 ug)	3-1 (500 ng)	3-2 (500 ng)	Full-length	Catalytic fragment
K_m _(μM)	193 ± 26	537 ± 14	445 ± 41	406 ± 11	263 ± 12	295 ± 22
K_cat _(s^-1^)	270 ± 10	118 ± 1.1	570 ± 19	612 ± 6.1	836 ± 11	1690 ± 39
K_cat_/K_m_	1.40	0.22	1.28	1.51	3.2	5.7

### Folding of CNPase

**Figure 4:**
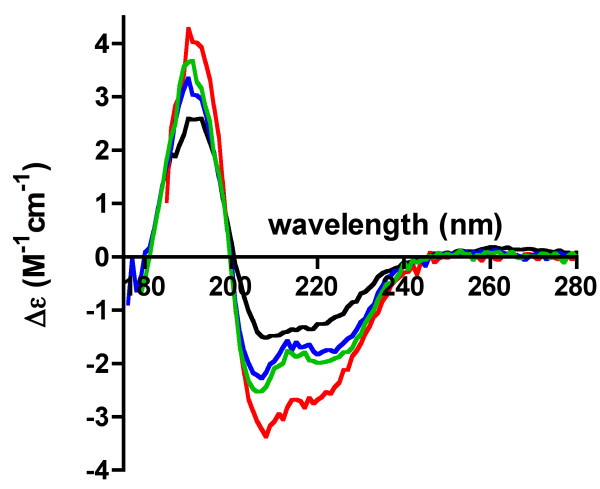
**Analysis of the folding of CNPase by SRCD**. The spectra are shown for the C-terminal domain (blue), N-terminal domain (black), and the full-length protein (red). In addition, the spectrum for the C-terminal domain in 1 M ammonium sulphate is shown (green).

The folding of 3 different CNPase constructs; the full-length protein (construct 2-1) and the isolated N- and C-terminal domains (constructs 4-3 and 6-1) was assessed by SRCD (Figure 4). The spectra indicate that all 3 tested samples were folded, containing both alpha and beta structure. The secondary structure contents obtained using Dichroweb are listed in Table 2. Notably, it is clear that the N-terminal domain has more β-sheet structure and less α-helices than the C-terminal domain. Upon comparison to the known crystal and solution structures of the catalytic domain, the spectrum for the C-terminal domain indicates a smaller content of helical segments than the 3D structures.

**Table 2:** Deconvolution of SRCD spectra

sample	helix	sheet
Full-length CNPase	28	23

N-terminal domain	8	39

C-terminal domain	16	33

Crystal structure (C-terminal)	30	32

NMR structure (C-terminal)	28	27

The spectra were analyzed at the Dichroweb server using the CDSSTR algorithm and the SP175 reference dataset.

## Conclusions

CNPase is an abundant myelin protein that was discovered already nearly 50 years ago [12], but whose actual function has only recently begun to be revealed. CNPase has been shown to bind RNA, and its physiological function could be related to RNA metabolism [14,15]. However, it is not known, how RNA is bound by CNPase, and why it happens. We have shown here that both the N- and C-terminal domain of recombinant CNPase are folded entities, and will start thorough studies on the functional properties and structure of these domains, both in isolation and together, in the context of full-length CNPase. Specifically, a thorough characterization of the interactions, structure, and function of the N-terminal domain of CNPase is expected to shed light on the function of the enzyme in myelin.

## Competing interests

The authors declare that they have no competing interests.

## Authors' contributions

MM carried out the experiments. PK collected SRCD data. Both authors conceived of the study and wrote the manuscript, and read and approved the final manuscript.

## Supplementary Material

Additional file 1 **Detailed methods for vector construction**. Detailed protocols for expression vector construction, including a table of the used primers (Supplementary Table S1). Click here for file

Additional file 2 **CNPase constructs**. A table describing all prepared expression constructs, related to the information shown on Figure 1 (Supplementary Table S2). Click here for file

Additional file 3 **Thermal shift assays**. Detailed conditions for thermal shift assays and additional results (Supplementary Table S3). Click here for file
