# Supplementary material for: Expression, purification, and initial characterization of different domains of recombinant mouse 2',3'-cyclic nucleotide 3'-phosphodiesterase, an enigmatic enzyme from the myelin sheath
Source: BMC Res Notes. 2010 Jan 21;3:12. doi: 10.1186/1756-0500-3-12 (PMC2843729; doi:10.1186/1756-0500-3-12)
Supplement: Additional file 1 — Detailed methods for vector construction. Detailed protocols for expression vector construction, including a table of the used primers (Supplementary Table S1). [file 1756-0500-3-12-S1.DOC]

# Supplementary File 1

### Methods for vector construction

Mouse CNP2 cDNA [26] was used as a template for the preparation of total of 18 expression clones using the Gateway method. The desired region of the cDNA was first multiplied through polymerase chain reaction (PCR) with appropriate primers (Eurofins MWG). The primers are listed below in Supplementary Table 1. The PCR reaction mixture contained 10 fmol of both forward and reverse primers, 1 µl of 1:1000 dilution of cDNA, 1 x Phusion buffer (Finnzymes), 4 fmol of dNTP, 2.5 % DMSO and 0.2 U of Phusion High-Fidelity DNA polymerase (Finnzymes) in a final volume of 20 µl. The success of the PCR reaction was checked by agarose gel electrophoresis, and the PCR products were purified using PEG precipitation: to 15 µl of PCR product, 45 µl of TE buffer and 30 µl of 30 % PEG 8000 / 30 mM MgCl_2_ were added, the mixture was vortexed and centrifuged for 15 minutes at 15000 rpm, and the pellet was redissolved in 15 µl of TE buffer.

To add the attB-cloning sites to the DNA constructs, a second PCR reaction was performed. The reaction mixture was identical except that 1 µl of the product of the first PCR was used instead of cDNA, and the forward and reverse primers were replaced with 16 fmol of attB1 and attB2 primers (Supplementary Table 1). The product DNA was analyzed and purified in a similar fashion as above.

The PCR products, now containing the cloning regions for Gateway cloning, were subjected to the BP-reaction to produce an entry clone for the Gateway protocol. 25 fmol of purified PCR product was used per reaction. The reaction mixture consisted of approximately 30 fmol of the donor vector pDONR221 (Invitrogen) and 1 µl of Gateway BP clonase II Enzyme mix (Invitrogen) in a volume of 5 µl in TE buffer. The reaction was carried out at 25 °C overnight, 0.5 µl of Proteinase K were added, and the samples were incubated at 37 °C for 10 min to destroy the clonase activity. The reaction product was transformed into DH5α-cells and plated onto LB plates containing 50 µg/ml kanamycin. The resulting colonies were analysed through colony-PCR, identical to the second PCR reaction described above, except that the template was derived from the colony that was being analysed. Colonies showing a single band of correct size in agarose gel electrophoresis were selected, and the plasmid was purified using the Nucleospin Plasmid Quickpure (Macherey-Nagel) or HiYield Plasmid Mini (Real Biotech Corp.) kits. The purified plasmids were sequenced.

Entry clones with correct sequences were subjected to the LR-reaction with the destination vector pTH27 [27] to produce expression clones coding for an N-terminal hexahistidine tag. A TEV protease cleavage site is introduced between the tag and the protein sequence in such a fashion, that only one extra glycine residue remains in the N-terminus of the recombinant protein after cleavage [28]. The LR reaction mixture contained approximately 25 fmol of entry clone, 7 fmol of pTH27 and 1 µl of Gateway LR Clonase II Enxyme Mix (Invitrogen) in a total volume of 5 µl in TE buffer. Reactions were incubated at 25 °C overnight, and Proteinase K was again used destroy the clonase activity as above. The reaction product was transformed into DH5α-cells and plated onto LB plates containing 100 µg/ml ampicillin. The resulting colonies were again analyzed by colony-PCR and sequenced. Expression clones containing the correct sequence were stored at -20 °C for further use.

| Supplementary Table 1. Primer sequences used in PCR reactions. | |
| --- | --- |
|  |  |
| Primer | Sequence |
| Forward 1 | GCTCTGAGAATCTTTATTTTCAGGGCATGAACACAAGCTTTACCCG |
| Forward 2 | GCTCTGAGAATCTTTATTTTCAGGGCGGAGCAAAGGAGAAGCCAG |
| Forward 3 | GCTCTGAGAATCTTTATTTTCAGGGCCTGCCACTCTACTTTGGC |
| Forward 4 | GCTCTGAGAATCTTTATTTTCAGGGCAAAATGTCATCCTCAGGAGC |
| Forward 5 | GCTCTGAGAATCTTTATTTTCAGGGCGAGCTGCAGTTCCCTTTCC |
| Forward 6 | GCTCTGAGAATCTTTATTTTCAGGGCGGGCTGGAGAAGGACTTTC |
| Forward 7 | GCTCTGAGAATCTTTATTTTCAGGGCGCCCAGCTCAAGGAGAAG |
|  |  |
| Reverse 1 | AGAAAGCTGGGTTCACCCATAGTACCCCGTGAAG |
| Reverse 2 | AGAAAGCTGGGTTCAGATGATGGTGCAGATCTGC |
| Reverse 3 | AGAAAGCTGGGTTCACAGCCCGGGCTTCAGC |
|  |  |
| attB1 | GGGGACAAGTTTGTACAAAAAAGCAGGCTCTGAGAATC |
| attB2 | GGGGACCACTTTGTACAAGAAAGCTGGGT |
