# Supplementary material for: Expression, purification, and initial characterization of different domains of recombinant mouse 2',3'-cyclic nucleotide 3'-phosphodiesterase, an enigmatic enzyme from the myelin sheath
Source: BMC Res Notes. 2010 Jan 21;3:12. doi: 10.1186/1756-0500-3-12 (PMC2843729; doi:10.1186/1756-0500-3-12)
Supplement: Additional file 2 — CNPase constructs. A table describing all prepared expression constructs, related to the information shown on Figure 1 (Supplementary Table S2). [file 1756-0500-3-12-S2.DOC]

| Supplementary Table 2. CNPase constructs. Residue numbers are from the sequence of mouse CNP2. | | | | |
| --- | --- | --- | --- | --- |
|
| Construct | First residue | Last residue | Length in residues | MW (kDa) |
| 1-1 | 1 | 398 | 398 | 44.9 |
| 1-2 | 1 | 420 | 420 | 47.2 |
| 1-3 | 1 | 180 | 180 | 20.9 |
| 2-1 | 25 | 398 | 374 | 42.0 |
| 2-2 | 25 | 420 | 396 | 44.3 |
| 2-3 | 25 | 180 | 156 | 18.0 |
| 3-1 | 185 | 398 | 214 | 23.6 |
| 3-2 | 185 | 420 | 236 | 25.9 |
| 4-1 | 20 | 398 | 379 | 42.6 |
| 4-2 | 20 | 420 | 401 | 44.9 |
| 4-3 | 20 | 180 | 161 | 18.6 |
| 5-1 | 31 | 398 | 368 | 41.4 |
| 5-2 | 31 | 420 | 390 | 43.7 |
| 5-3 | 31 | 180 | 150 | 17.4 |
| 6-1 | 179 | 398 | 220 | 24.2 |
| 6-2 | 179 | 420 | 242 | 26.5 |
| 7-1 | 158 | 398 | 241 | 26.7 |
| 7-2 | 158 | 420 | 263 | 29.0 |
