# Supplementary material for: Expression, purification, and initial characterization of different domains of recombinant mouse 2',3'-cyclic nucleotide 3'-phosphodiesterase, an enigmatic enzyme from the myelin sheath
Source: BMC Res Notes. 2010 Jan 21;3:12. doi: 10.1186/1756-0500-3-12 (PMC2843729; doi:10.1186/1756-0500-3-12)
Supplement: Additional file 3 — Thermal shift assays. Detailed conditions for thermal shift assays and additional results (Supplementary Table S3). [file 1756-0500-3-12-S3.DOC]

| Supplementary Table 3. T_m_-values from thermal shift assays. | | | | |  |  |  |  |
| --- | --- | --- | --- | --- | --- | --- | --- | --- |
|  |  |  |  |  |  |  |  |  |
| Assay 1 - C-terminal CNP | | | | |  |  |  |  |
|  | pH | | | |  |  |  |  |
| Additive | 5.5 | 6.5 | 7.5 | 8.5 |  |  |  |  |
| None | 67 | 66 | 64 | 62 |  |  |  |  |
| 150 mM NaCl | 67 | 66 | 65 | 63 |  |  |  |  |
| 500 mM NaCl | 67.5 | 67 | 66 | 64.5 |  |  |  |  |
| 10 % Glycerol | 67 | 66.5 | 65 | 63 |  |  |  |  |
| 30 % Glycerol | 67 | 66.5 | 66 | 64 |  |  |  |  |
| 50 mM Arginine & 50 mM Glutamine | 67 | 67 | 66 | 65.5 |  |  |  |  |
| 10 mM MgSO4 | 67 | 66 | 65.5 | 65 |  |  |  |  |
| 50 mM Glycine | 67 | 66 | 66 | 65 |  |  |  |  |
| 10 mM Glucose | 67 | 66 | 65 | 63 |  |  |  |  |
| 10 mM CaCl2 | 67 | 66 | 65.5 | 65 |  |  |  |  |
| 5 mM 2'-3'-cAMP | 67 | 66.5 | 65 | 64 |  |  |  |  |
| 1 mM EDTA | 67 | 66 | 65 | 63.5 |  |  |  |  |
|  |  |  |  |  |  |  |  |  |
| Assay 1 - Full-length CNP | | | | |  |  |  |  |
|  | pH | | | |  |  |  |  |
| Additive | 5.5 | 6.5 | 7.5 | 8.5 |  |  |  |  |
| None | 64 | 61.5 | 60 | 55 |  |  |  |  |
| 150 mM NaCl | 64 | 62 | 60 | 56.5 |  |  |  |  |
| 500 mM NaCl | 65 | 63 | 59 | 57 |  |  |  |  |
| 10 % Glycerol | 64 | 62.5 | 60 | 57 |  |  |  |  |
| 30 % Glycerol | 64.5 | 61.5 | 60.5 | 58.5 |  |  |  |  |
| 50 mM Arginine & 50 mM Glutamine | 64 | 63 | 61 | 61 |  |  |  |  |
| 10 mM MgSO4 | 64 | 62 | 64.5 | 60 |  |  |  |  |
| 50 mM Glycine | 64 | 63 | 61 | 60 |  |  |  |  |
| 10 mM Glucose | 64 | 62 | 59.5 | 56.5 |  |  |  |  |
| 10 mM CaCl2 | 64 | 63 | 61 | 60 |  |  |  |  |
| 5 mM 2'-3'-cAMP | 65 | 62.5 | 59.5 | 58.5 |  |  |  |  |
| 1 mM EDTA | 64 | 62.5 | 61 | 59 |  |  |  |  |
|  |  |  |  |  |  |  |  |  |
| Assay 2 - C-terminal CNP | | | | |  |  |  |  |
|  | pH | | | | | | | |
| Additive | 3.5 | 4 | 4.5 | 5 | 5.5 | 6 | 6.5 | 7.5 |
| None | 61 | 64 | 67.5 | 67 | 67 | 65.5 | 65 | 63 |
| 150 mM NaCl | 57 | 61 | 65 | 66.5 | 67 | 66 | 66 | 64 |
| 500 mM NaCl | 56 | 59 | 64 | 66 | 68 | 68 | 68 | 66 |
| 10 % Glycerol | 58 | 61 | 65 | 67 | 68 | 66.5 | 66 | 65 |
| 5 % PEG 400 | 56.5 | 59.5 | 63.5 | 65 | 65.5 | 64.5 | 64 | 62 |
| 5 % PEG 2000 | 57 | 61 | 65 | 66 | 67 | 66 | 66 | 64 |
